# Genome analysis of “*Candidatus* Aschnera chinzeii,” the bacterial endosymbiont of the blood-sucking bat fly *Penicillidia jenynsii* (Insecta: Diptera: Nycteribiidae)

**DOI:** 10.3389/fmicb.2023.1336919

**Published:** 2024-01-22

**Authors:** Ryuichi Koga, Minoru Moriyama, Tomonari Nozaki, Takema Fukatsu

**Affiliations:** ^1^Bioproduction Research Institute, National Institute of Advanced Industrial Science and Technology (AIST), Tsukuba, Japan; ^2^Department of Biological Sciences, Graduate School of Science, The University of Tokyo, Tokyo, Japan; ^3^Graduate School of Life and Environmental Sciences, University of Tsukuba, Tsukuba, Japan

**Keywords:** Aschnera chinzeii, nycteribiid bat fly, *Penicillidia jenynsii*, symbiotic bacteria, genome reduction, B vitamin provisioning, blood-sucking insect

## Abstract

Insect–microbe endosymbiotic associations are omnipresent in nature, wherein the symbiotic microbes often play pivotal biological roles for their host insects. In particular, insects utilizing nutritionally imbalanced food sources are dependent on specific microbial symbionts to compensate for the nutritional deficiency via provisioning of B vitamins in blood-feeding insects, such as tsetse flies, lice, and bedbugs. Bat flies of the family Nycteribiidae (Diptera) are blood-sucking ectoparasites of bats and shown to be associated with co-speciating bacterial endosymbiont “*Candidatus* Aschnera chinzeii,” although functional aspects of the microbial symbiosis have been totally unknown. In this study, we report the first complete genome sequence of *Aschnera* from the bristled bat fly *Penicillidia jenynsii*. The *Aschnera* genome consisted of a 748,020 bp circular chromosome and a 18,747 bp circular plasmid. The chromosome encoded 603 protein coding genes (including 3 pseudogenes), 33 transfer RNAs, and 1 copy of 16S/23S/5S ribosomal RNA operon. The plasmid contained 10 protein coding genes, whose biological function was elusive. The genome size, 0.77 Mbp, was drastically reduced in comparison with 4–6 Mbp genomes of free-living γ-proteobacteria. Accordingly, the *Aschnera* genome was devoid of many important functional genes, such as synthetic pathway genes for purines, pyrimidines, and essential amino acids. On the other hand, the *Aschnera* genome retained complete or near-complete synthetic pathway genes for biotin (vitamin B7), tetrahydrofolate (vitamin B9), riboflavin (vitamin B2), and pyridoxal 5'-phosphate (vitamin B6), suggesting that *Aschnera* provides these vitamins and cofactors that are deficient in the blood meal of the host bat fly. Similar retention patterns of the synthetic pathway genes for vitamins and cofactors were also observed in the endosymbiont genomes of other blood-sucking insects, such as *Riesia* of human lice, *Arsenophonus* of louse flies, and *Wigglesworthia* of tsetse flies, which may be either due to convergent evolution in the blood-sucking host insects or reflecting the genomic architecture of *Arsenophonus*-allied bacteria.

## Introduction

Insect–microbe endosymbiotic associations are omnipresent in nature, wherein the symbiotic microbes often play pivotal biological roles for their host insects (Buchner, 1965; Bourtzis and Miller, 2003). In particular, insects utilizing nutritionally imbalanced food sources are dependent on specific microbial symbionts to compensate for the nutritional deficiency via provisioning of essential amino acids in plant sap-feeding insects, such as aphids, leafhoppers, and cicadas (Moran et al., 2008; Douglas, 2009), or supply of B vitamins in blood-feeding insects, such as tsetse flies, lice, and bedbugs (Rio et al., 2016; Husnik, 2018).

Vertebrate blood is certainly nutritious, which comprises a rich protein source mainly in the form of hemoglobin, but it entails disproportionally low levels of lipids and carbohydrates, high levels of iron that is potentially toxic, and deficiency in some important nutritional elements such as B vitamins (Sterkel et al., 2017). Therefore, many insects and other invertebrates nutritionally dependent solely on vertebrate blood all through their life stages, including tsetse flies, louse flies, bat flies, lice, bedbugs, kissing bugs, ticks, and leeches, are obligatorily associated with specific symbiotic bacteria whose main biological roles are suggested as provisioning of B vitamins (Rio et al., 2016; Husnik, 2018; Duron and Gottlieb, 2020; Fukatsu et al., 2023).

**Figure 1:**
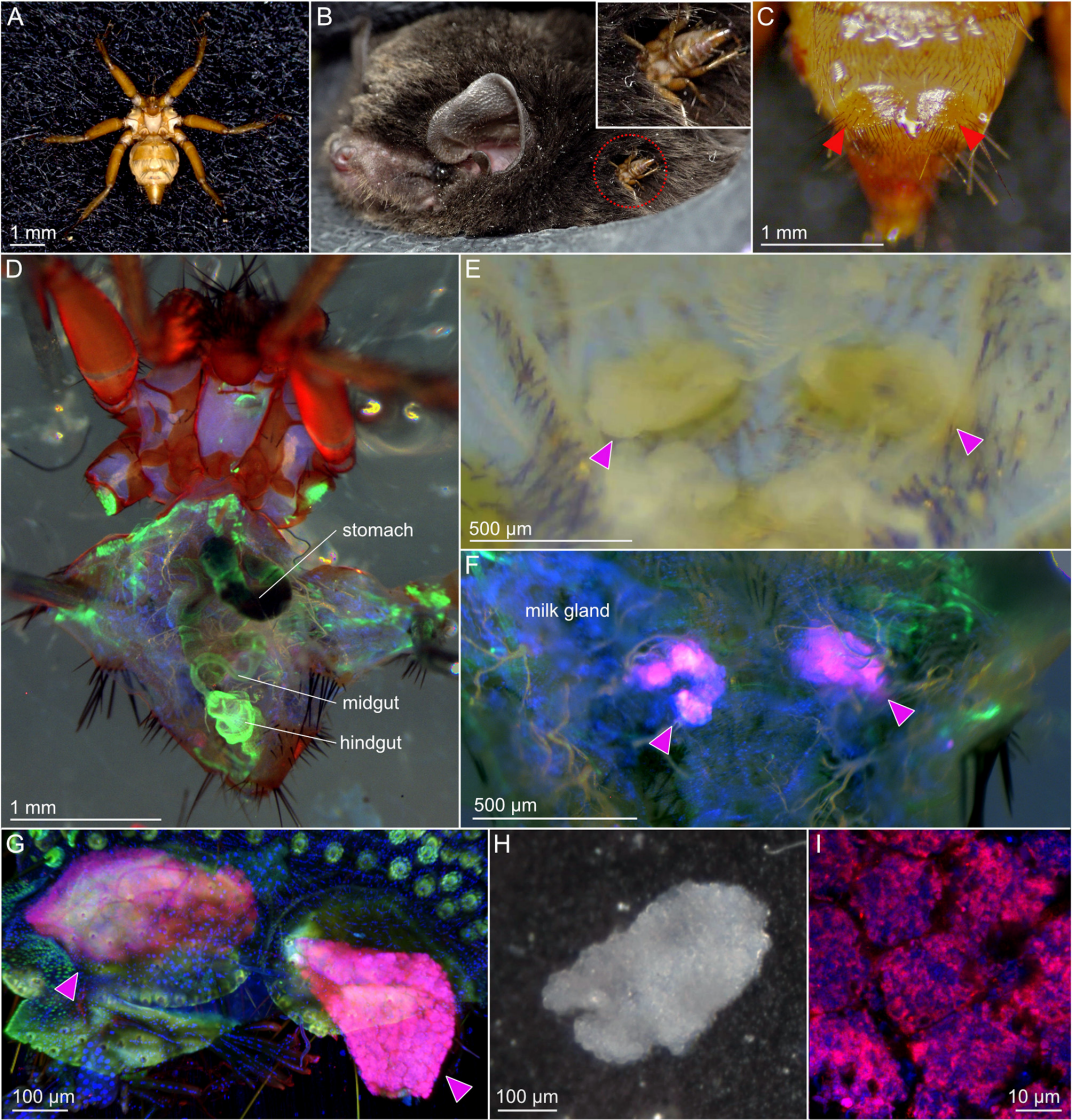
The bat fly *Penicillidia jenynsii* and its *Aschnera*-harboring bacteriomes. **(A)** Dorsal view of a female insect. **(B)** An insect parasitizing the host bat *Miniopterus fuliginosus*. **(C)** Ventral view of a female insect's abdomen, on which the projections housing the bacteriomes inside are seen (arrowheads). **(D)** Dorsal view of a female insect whose abdomen is cut open at the dorsal midline. Internal organs, such as stomach, midgut and hindgut are seen. **(E)** Enlarged bright-field image of the abdomen from which the internal organs are removed. Whitish bacteriomes packed inside the projections on the ventral plate are exposed (arrowheads). **(F)** Fluorescent image of the abdomen from which the internal organs are removed. Strong bacterial signals due to *Aschnera* localization are detected in the bacteriomes (arrowheads). **(G)** Enlarged fluorescent image of the bacteriomes to which *Aschnera* is localized (arrowheads). **(H)** Bright-field image of an isolated bacteriome. **(I)** Confocal fluorescent microscopic image of an isolated bacteriome in which *Aschnera* cells are visualized. In **(D**, **F**, **G**, **I)**, magenta, blue, and green indicate Alexa Fluor 555-labeled FISH signals of bacterial 16S rRNA, DAPI signals of DNA, and Alexa Fluor 488 phalloidin signals of filamentous actin, respectively.

Bat flies of the family Nycteribiidae (Diptera) are blood-sucking ectoparasites of bats of peculiar spider-like appearance with highly specialized morphological traits, represented by lack of wings, reduced head and eyes, long and powerful legs with grasping claws, and flexible and tough exoskeleton (Figures 1A, B). The nycteribiid bat flies (Nycteribiidae) constitute the superfamily Hippoboscoidea together with tsetse flies (Glossinidae), louse flies (Hippoboscidae), and streblid bat flies (Streblidae) (Petersen et al., 2007), which share such remarkable traits as adult feeding solely on vertebrate blood, adenotrophic viviparity with highly developed uterus and milk glands, and larval uterine growth with no free life until pupation outside the maternal body (Lehane, 2005; Dick and Patterson, 2006; Nikoh et al., 2011). Reflecting their obligatory blood-feeding lifestyle, these insect groups rely on specific symbiotic bacteria and develop specialized cells and organs, called bacteriocytes and bacteriomes, for retaining the microbial partners (Roubaud, 1919; Zacharias, 1928; Aschner, 1931; Buchner, 1965). All tsetse flies are associated with the monophyletic endosymbiotic bacteria *Wigglesworthia glossinidea* (Aksoy, 2000; Rio et al., 2012; Wang et al., 2013). All nycteribiid bat flies are associated with and co-speciating with the monophyletic endosymbiotic bacteria “Candidatus *Aschnera chinzeii*” (hereafter called *Aschnera*) (Hosokawa et al., 2012; Duron et al., 2014). Among louse flies, their *Arsenophonus* endosymbionts do not necessarily exhibit host-symbiont co-speciation and are thought to be of multiple evolutionary origins (Dale et al., 2006; Trowbridge et al., 2006; Duron et al., 2014; Nováková et al., 2015; Ríhová et al., 2023). As for streblid bat flies, while an array of bacterial associates has been identified, the main symbiotic bacteria are elusive (Trowbridge et al., 2006; Morse et al., 2012, 2013).

Except for several tsetse species (Mews et al., 1977), laboratory rearing and experiments are very challenging for the blood-sucking ectoparasitic flies. Hence, to gain insight into functional aspects of the symbiotic bacteria, their genomic information is very important. As for the Glossinidae, the *Wigglesworthia* genomes have been determined for the tsetse flies *Glossinidia brevipalpis* (Akman et al., 2002) and *G. morsitans* (Rio et al., 2012). As for the Hippoboscidae, the genomes of *Arsenophonus melophagi* from the sheep ked *Melophagus ovinus* (Nováková et al., 2015) and *Arsenophonus lipoptenae* from the deer louse fly *Lipoptena cervi* (Nováková et al., 2016) have been reported. As for the Nycteribiidae, no symbiont genome has been reported thus far.

In this study, we report the first genome sequencing and analysis of *Aschnera* endosymbiont of the bat fly *Penicillidia jenynsii* parasitizing the bat *Miniopterus fuliginosus*.

## Materials and methods

### Insect materials

The bat flies *P. jenynsii* were collected from the host eastern bent-winged bats *M. fuliginosus* caught at Kawabe County, Hyogo Prefecture, Japan, on 14 May and 16 September 2022 in field bat monitoring conducted by Nobutaka Urano and Shunji Fujita (permission number 環近地野許第 2107301 号, the Ministry of the Environment, Japan). The collected bat flies were placed in plastic tubes with wet tissue paper, kept in a cooling box, brought to laboratory, and used for experiments.

### Dissection and DNA extraction

Each adult female *P. jenynsii* was fixed to a dissection stage assembled with a silicon rubber disk and a plastic Petri dish by pinning at the center of its flat thorax. Legs of the insect were removed using ophthalmological microscissors in a phosphate buffered saline (PBS: 0.8% NaCl, 0.02% KCl, 0.115% Na_2_HPO_4_, 0.02% KH_2_PO_4_, pH 7.4), and their dorsal abdomen was cut open along the midline to expose the internal organs of the insect. Fat bodies, milk glands, and digestive tract were all carefully removed not to break the stomach full of blood meal, by which whitish bacteriomes packed in the paired projections on the fifth ventral plate were exposed and carefully collected using fine forceps. In total, 60 bacteriomes from 30 insects were collected and subjected to DNA extraction using a DNA tissue mini kit (Qiagen).

### Genome sequencing and assembly

The DNA sample extracted from the bacteriomes was subjected to the library preparation for long-read sequencing by using Ligation sequencing kit SQK-LSK109 (Oxford Nanopore Technologies). The library prepared was analyzed on a MinION sequencer implemented with the flow cell R9.4.1 (Oxford Nanopore Technologies). The DNA sample was also subjected to short-read sequencing. The short-read library was prepared using NEBNext Ultra II DNA Library Prep Kit for Illumina (New England Biolab), and 150 bp paired-end reads were obtained by NovaSeq 6000 (Illumina). Because excessive short-read data were obtained, a 10% subset was extracted from the original read library. Furthermore, the subset was quality-trimmed on CLC genomics workbench version 23 (Qiagen). A short- and long-read hybrid assembling was conducted by using Unicycler version 0.5.0 (Wick et al., 2017). Assemble errors in the resultant draft genome sequence were searched and manually corrected with the assistance of CLC genomics workbench. The circularity of the assembled genome was confirmed by mapping the long reads to the rotated contigs and checking the resultant read maps. Annotation of the corrected genome was performed using DFAST version 1.2.18 (Tanizawa et al., 2018). The BUSCO scores were calculated with the enterobacterales_odb10 database using BUSCO version 5.5.0 (Manni et al., 2021). The genome map was drawn using DNAplotter in Artemis version 18.0.0 (Carver et al., 2009).

### Phylogenetic analysis

Deduced protein sequences inferred from the *Aschnera* genome and 31 bacterial genomes retrieved from DNA databases (GenBank, accessed in July, 2023) were prepared from the annotated genome sequences using CLC genomic workbench. BCGtree pipeline (Ankenbrand and Keller, 2016) was used to assemble and concatenate the multiple alignments of 107 bacterial core protein genes from 32 bacterial genomes with the following parameter: –min-proteomes = 2. The resultant concatenated alignment and corresponding partition information were used to infer the maximum likelihood tree with 1,000 bootstrap replicates using IQ-TREE version 2.2.2.7 (Minh et al., 2020) with the following parameter: -m MFP+MERGE. The phylogenetic tree was visualized using TreeViewer version 2.1.0 (Bianchini and Sánchez-Baracaldo, 2023).

### Metabolic pathway analysis

The metabolic capability of *Aschnera* was predicted based on the genome sequence using GhostKOALA web service (https://www.kegg.jp/ghostkoala/) (Kanehisa et al., 2016). The metabolic models of the endosymbiont of human louse, *Riesia pediculicola* strain USDA (T number of the KEGG database: T01218), the endosymbiont of louse fly *Arsenophonus lipoptenae* strain CB (T04248), the endosymbiont of tsetse fly *Wigglesworthia glossinidia* (T00101), the endosymbiont of bedbug *Wolbachia* sp. strain *w*Cle (T03628), the endosymbiont of aphid *Buchnera aphidicola* strain APS (T00036), the gut symbiont of plataspid stinkbug *Ishikawaella capsulata* (T04149), and *Escherichia coli* strain K-12 MG1655 (T00007) were acquired from the KEGG database (https://www.kegg.jp/) (Kanehisa et al., 2022). The metabolic pathways were compared essentially based on KEGG modules with references to the EcoCyc database (Keseler et al., 2021).

### Whole-mount fluorescence *in situ* hybridization (wFISH)

The abdomen of each adult female *P. jenynsii* was cut to make a slit at the midline using ophthalmological microscissors in PBS. Then, the insects were fixed in PBS containing 4% paraformaldehyde at 4°C overnight. After several washes with PBS, the specimens were subjected to wFISH as previously described (Koga et al., 2009). The specimens were hybridized with Al555-EUB338 (5'-Alexa Fluor 555- GCT GCC TCC CGT AGG AGT-3') targeting bacterial 16S ribosomal RNA. After overnight hybridization at room temperature, the specimens were washed with PBT (PBS containing 0.1% Tween-20) containing 165 nM Alexa Fluor 488 phalloidin and 2 μg/mL 4',6-diamidino-2-phenylindole (DAPI) for 30 min at room temperature and subsequently washed with PBT three times. The hybridized specimens were dissected and pictured in PBS under a fluorescence dissection microscope M165 controlled by LAS version 4.13.0 (Leica). The bacteriomes on the abdominal cuticle were mounted in PBS-50% glycerol and observed with a laser scanning microscope LSM700 controlled with Zen 2011 version 7.0 (Zeiss). Digital images were manually manipulated using Affinity Photo version 2.1.1 (Affinity).

## Results and discussion

### Endosymbiotic system of *P. jenynsii*

The bat fly *P. jenynsii* (Figure 1A) is a blood-sucking ectoparasite of the eastern bent-winged bat *M. fuliginosus* (Figure 1B). On the ventral side of the abdomen of a female insect, the fifth ventral plate bears paired projections on both sides (Figure 1C), each of which contains the bacteriome consisting of the bacteriocytes harboring the symbiotic bacteria (Hosokawa et al., 2012). When the abdomen of a female insect was dissected from the dorsal side (Figure 1D) and deprived of the internal organs (alimentary tract, gonad, fat body, etc.), the bacteriome was seen as a whitish cell mass inside each of the projections (Figure 1E). FISH targeting bacterial 16S rRNA detected the symbiont signals within the bacteriomes in the abdominal projections (Figures 1F, G). The bacteriomes were carefully isolated (Figure 1H), wherein the symbiotic bacteria within numerous bacteriocytes were visualized using FISH (Figure 1I).

### Genome sequence of *Aschnera*

**Figure 2:**
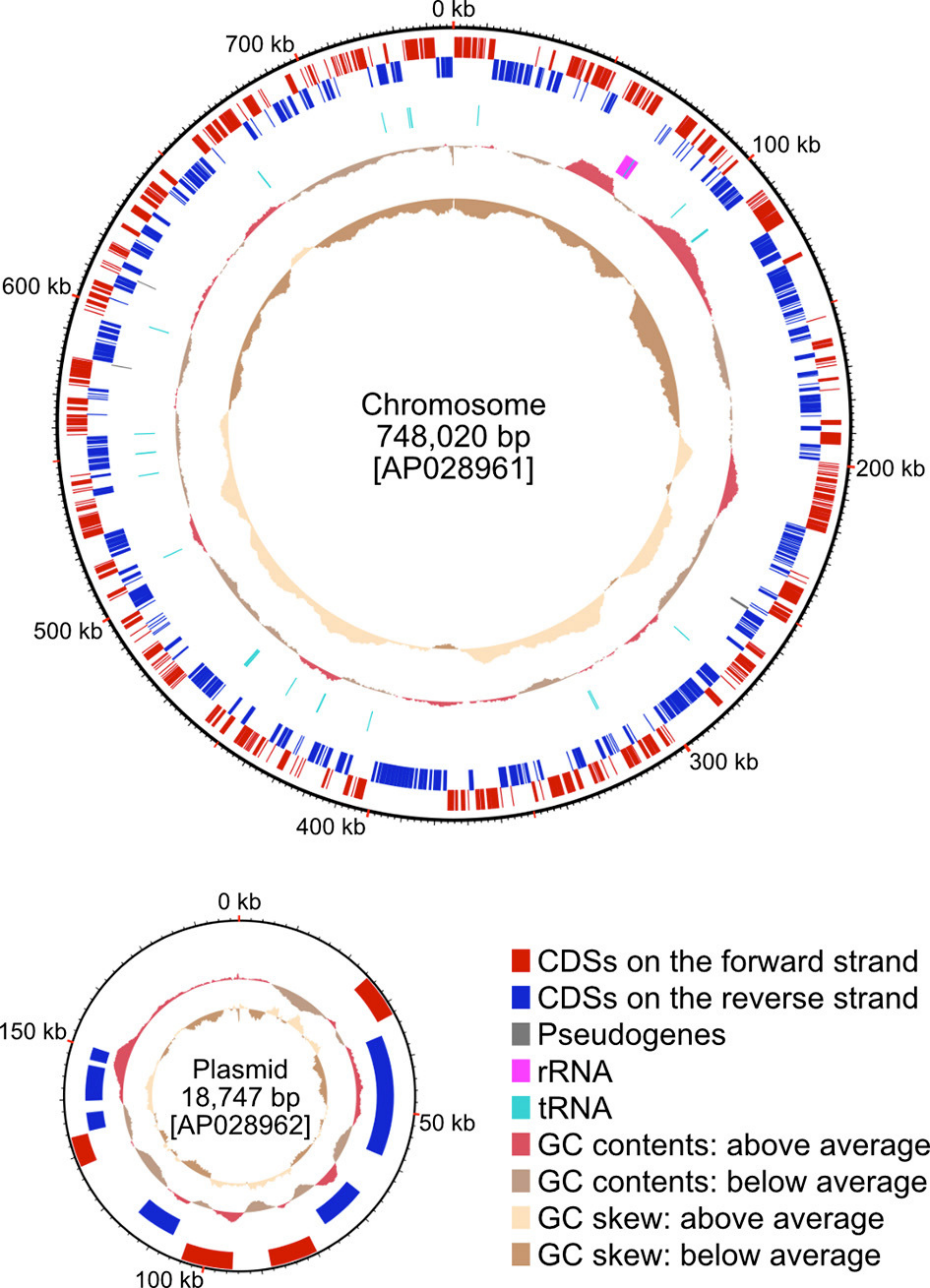
The genome map of *Aschnera*. The chromosome is oriented to make the reading frame of *dnaA* gene the first CDS on the forward strand. The plasmid is oriented arbitrarily. The outer-most circle represents the positions of CDSs on the forward strand while the second circle represents those on the reverse strand. The third circle represents the position of three pseudogenes. The fourth circle represents the positions of ribosomal and transfer RNA genes. Red, blue, gray, magenta, and cyan boxes represent CDSs on the forward strand, CDSs on the reverse strand, pseudogenes, and RNA-coding genes, respectively. The fifth and sixth circles represent GC contents and GC skews, respectively.

The isolated bacteriomes collected from 30 adult female *P. jenynsii* were subjected to DNA extraction, library construction, long- and short-read DNA sequencing, and genome assembly, whereby the complete *Aschnera* genome was determined as a 748,020 bp circular chromosome and a 18,747 bp circular plasmid. The latter was identified as the plasmid because of the presence of the replication initiation protein A (*repA*) gene in this contig. The genome size approximately agreed with a previous estimation of the genome size of *Aschnera* as 0.76 Mbp by pulsed field gel electrophoresis (Hosokawa et al., 2012). The 0.75 Mbp chromosome, consisting of 23.1% G + C, encoded 603 putative protein-coding sequences (CDSs), of which 3 were predicted as pseudogenes, 33 transfer RNAs, and 1 copy of 16S/23S/5S ribosomal RNA operon (Figure 2; Table 1; Supplementary Table S1). The 18 kbp plasmid, consisting of 22.3% G + C, encoded only 10 genes, whose biological relevance was elusive (Figure 2; Table 1; Supplementary Table S2). The *Aschnera* genome, 0.77 Mbp in size, is around 1/5–1/7 of the *E. coli* genomes of 4–6 Mbp (Lukjancenko et al., 2010). The BUSCO score of *A. chinzeii* was 81.1%. This score was higher than those of *R. pediculicola* USDA (66.8%) and *R*. sp. GBBU (61.8%). These results confirmed that *Aschnera* has experienced reductive genome evolution in the process of intimate co-evolution with nycteribiid bat flies (Hosokawa et al., 2012).

**Table 1:** Summary of the *Aschnera* genome sequencing and annotation.

	**Length (bp)**	**GC contents (%)**	**CDSs (Pseudogenes)**	**Average protein size (amino acids)**	**rRNA**	**tRNA**
Chromosome	748,020	23.1	600 (3)	328.62	3	33
Plasmid	18,747	22.3	10	282.2	0	0
Total	766,767	23.1	610 (3)	327.86	3	33

### Phylogenomic placement of *Aschnera*

**Figure 3:**
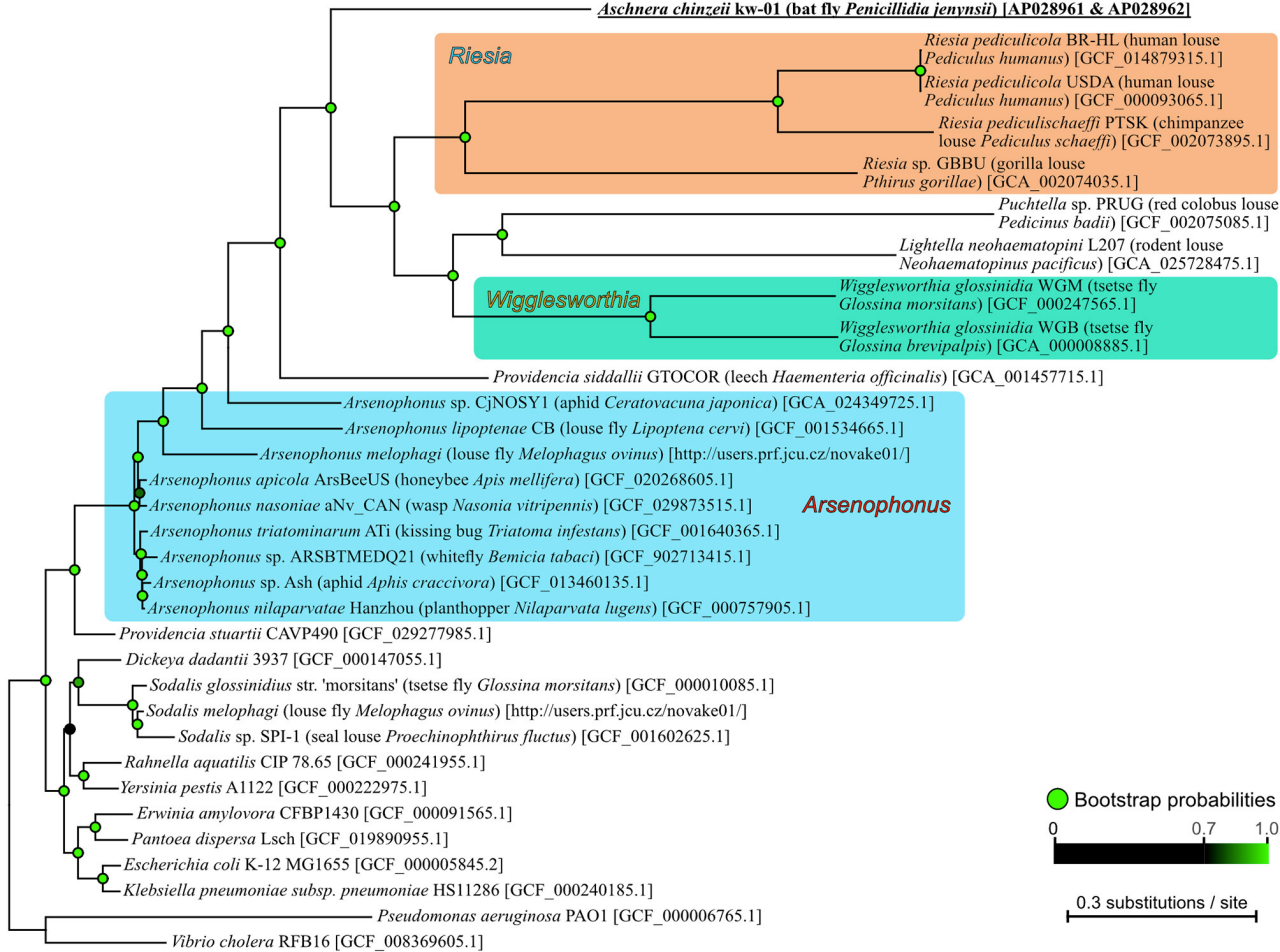
Phylogenetic placement of *Aschnera* in the γ-Proteobacteria. The maximum likelihood phylogeny was inferred from 33,136 amino acid sites inferred from concatenated 107 bacterial core protein genes. Generic, specific, and strain names of the bacteria are indicated on the right side of the phylogeny with host names in parentheses and sequence accession numbers in brackets. The bootstrap probabilities of the nodes were obtained from 1,000 resamplings, which were shown by color scale in the figure.

Molecular phylogenetic analysis based on 109 bacterial core protein sequences revealed that *Aschnera* is placed in the γ-proteobacterial clade consisting of *Arsenophonus* and allied bacterial lineages that embraces many insect-associated endosymbiotic bacteria (Nováková et al., 2009) (Figure 3). Among them, *Aschnera* was closely related to the endosymbionts of primate lice *Riesia* spp. (Kirkness et al., 2010; Boyd et al., 2014) and other endosymbionts of blood-sucking insects, such as *Lightella* of rodent lice (Ríhová et al., 2022), *Puchtella* of monkey lice (Boyd et al., 2017), and *Wigglesworthia* of tsetse flies (Akman et al., 2002; Rio et al., 2012). *Arsenophonus* endosymbionts of louse flies (Nováková et al., 2016) were placed outside of them in the phylogenetic tree. These phylogenetic patterns strongly suggested that although tsetse flies (Glossinidae), louse flies (Hippoboscidae), and bat flies (Nycteribiidae) belong to the same well-defined superfamily Hippoboscoidea (Petersen et al., 2007), their current endosymbiotic bacterial associates are likely of independent evolutionary origins presumably via symbiont acquisitions and replacements, as previously suggested (Nováková et al., 2009; Hosokawa et al., 2012; Duron et al., 2014; Ríhová et al., 2023).

### Gene repertoire of *Aschnera*

**Figure 4:**
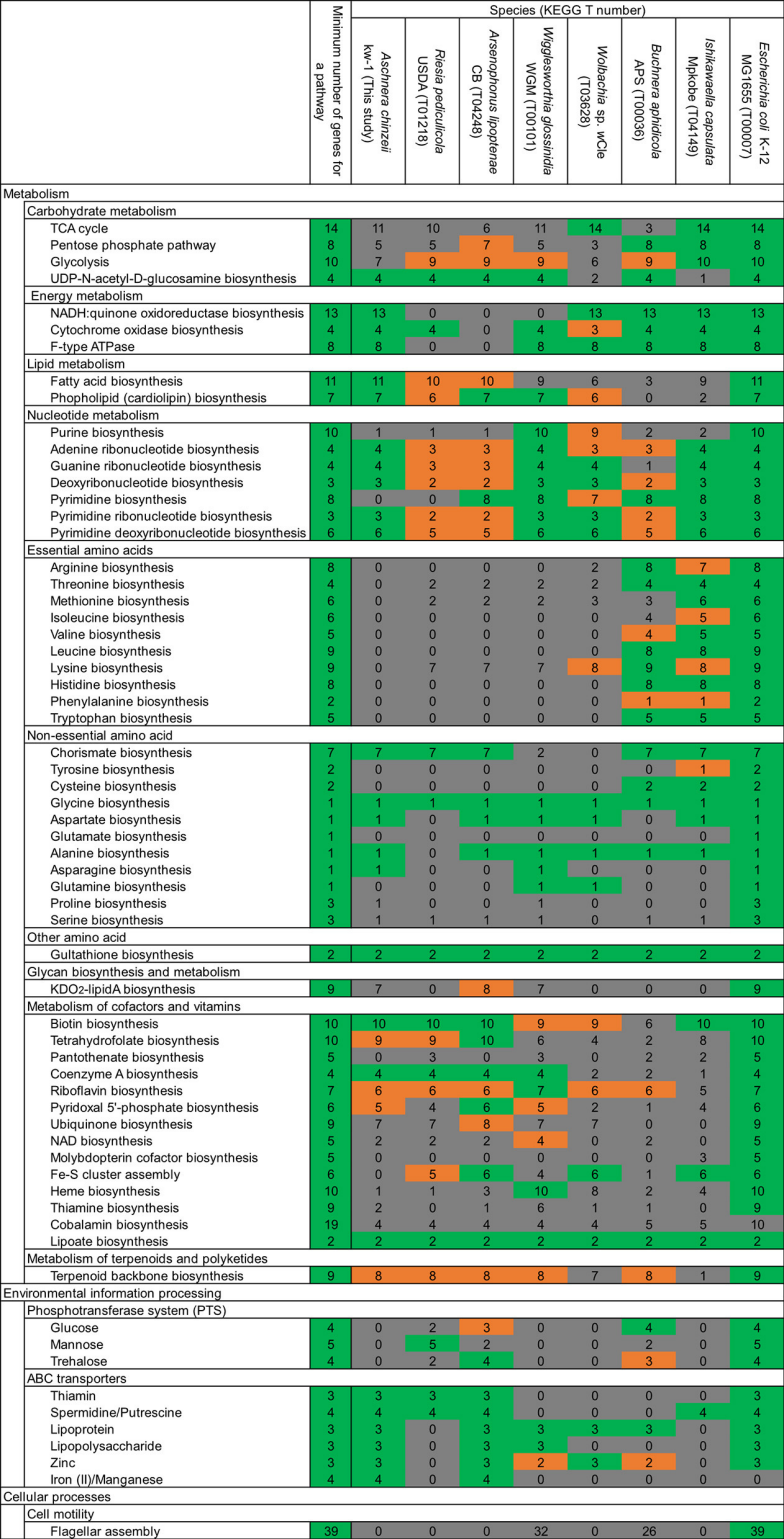
Comparison of metabolic gene repertoire between the *Aschnera* genome and other reduced endosymbiont genomes associated with blood-sucking and plant-sucking insects. The typical number of genes for each metabolic pathway is shown on the left side, whereas number of genes for each metabolic pathway encoded in the *E. coli* genome is shown on the right side. Colors indicate the completeness of the pathway in the genomes: green, complete; orange, 1 gene missing; gray, 2 or more genes missing.

In accordance with the drastic genome reduction, the *Aschnera* genome has lost many important metabolic pathways (Figure 4). For example, synthetic pathway genes for purines and pyrimidines are mostly missing. Strikingly, synthetic pathway genes for essential amino acids are completely lost from the *Aschnera* genome, which was also observed in the primary endosymbionts of other blood-sucking insects, including *Riesia* of human louse, *Arsenophonus* of louse fly, *Wigglesworthia* of tsetse fly, and *Wolbachia* of bedbug (Figure 4). These patterns may be relevant to the feeding habit of the host bat fly *P. jenynsii* as an obligatory blood-sucking ectoparasite of bats (Funakoshi, 1977; Lehane, 2005), considering that the blood meal is very rich in proteins and nucleic acids.

### Synthetic pathways for B vitamins in *Aschnera* genome

**Figure 5:**
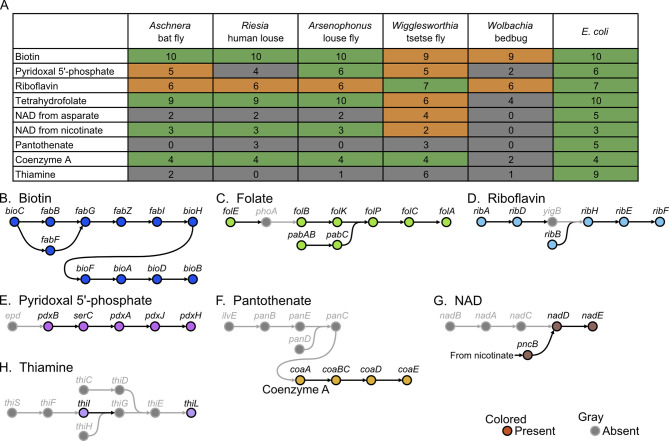
Synthesis pathway genes for B vitamins and cofactors encoded in the *Aschnera* genome. **(A)** Comparison of synthetic capabilities of B vitamins and other cofactors among *Aschnera* and other endosymbionts of blood-feeding insects. The typical number of genes for each metabolic pathway is shown on the right side represented by *E. coli*. Colors indicate the completeness of the pathway in the genomes: green, complete; orange, 1 gene missing; gray, 2 or more genes missing. **(B–H)** Presence and absence of genes in the synthesis pathways for B vitamins and cofactors. **(B)** Biotin (vitamin B7). **(C)** Tetrahydrofolate (vitamin B9). **(D)** Riboflavin (vitamin B2). **(E)** Pyridoxal 5'-phosphate (vitamin B6). **(F)** Coenzyme A (from pantothenate). **(G)** Nicotinamide adenine dinucleotide (NAD) (from nicotinate). **(H)** Thiamine (vitamin B1).

On the other hand, the blood meal is deficient in some nutrients, such as B vitamins. Therefore, obligatory blood-feeding insects, such as tsetse flies, lice, and bedbugs, rely on their specific bacterial symbiont for supply of B vitamins (Akman et al., 2002; Kirkness et al., 2010; Nikoh et al., 2014). Consequently, such symbiont genomes retain the synthetic pathway genes for some, if not all, vitamins and cofactors (Rio et al., 2016; Husnik, 2018; Duron and Gottlieb, 2020; Fukatsu et al., 2023). The *Aschnera* genome retained complete or near-complete synthetic pathway genes for biotin (vitamin B7), tetrahydrofolate (vitamin B9), riboflavin (vitamin B2), and pyridoxal 5'-phosphate (vitamin B6) (Figures 5A–E). The *Aschnera* genome also encoded synthetic pathway genes for such cofactors as coenzyme A (from pantothenate) and nicotinamide adenine dinucleotide (NAD) (from nicotinate) (Figures 5A, F, G). On the other hand, the *Aschnera* genome was incapable of synthesizing thiamine (vitamin B1) and pantothenate (vitamin B5) (Figures 5A, F, H). Based on these results, it seems plausible that *Aschnera* provides the host insect with biotin, pyridoxal, riboflavin, and tetrahydrofolate to compensate for nutritional deficiency of the blood meal. It is notable that not only *Aschnera* of the bat fly but also *Riesia* of human lice, *Arsenophonus* of louse flies, and *Wigglesworthia* of tsetse flies exhibit similar retention patterns of the synthesis pathway genes for B vitamins (Figure 5A), which may be either due to convergent evolution in the blood-sucking host insects or reflecting the genomic architecture of γ -proteobacterial *Arsenophonus*-allied bacteria. In this context, it seems meaningful that the α-proteobacterial mutualistic *Wolbachia* endosymbiont of bedbug retains synthesis pathway genes for biotin and riboflavin only (Figure 5A).

## Conclusion

In this study, we reported the complete genome sequence of *Aschnera*, the obligatory bacterial endosymbiont of the bat fly, and demonstrated that the symbiont genome has been streamlined for provisioning of B vitamins and cofactors that are deficient in the blood meal of the host bat fly. The bat fly is a very peculiar and experimentally intractable insect, being capable of surviving only on the body surface of specific bat species. Both the bat fly and the host bat are very difficult, or practically almost impossible, to maintain in the laboratory for experimental purposes. Therefore, the approach from the symbiont genomics provides invaluable information on the nature of the intimate microbial endosymbiosis in the non-model blood-sucking insect group Nycteribiidae. Transcriptomic, proteomic, and metabolomic approaches would further contribute to understanding the physiological, metabolic, and molecular interactions ongoing in the bat fly endosymbiosis.

## Data availability statement

The datasets presented in this study can be found in online repositories. The names of the repository/repositories and accession number(s) can be found below: https://ddbj.nig.ac.jp/search/en, DRA017199, DRA017200, AP028961, AP028962, SAMD00647857, SAMD00647858, PRJDB16712.

## Author contributions

RK: Conceptualization, Formal analysis, Investigation, Visualization, Writing – original draft, Writing – review & editing. MM: Investigation, Writing – review & editing. TN: Investigation, Writing – review & editing. TF: Conceptualization, Funding acquisition, Project administration, Supervision, Writing – original draft, Writing – review & editing.
